# Characteristics and Effects of Home-Based Digital Health Interventions on Functional Outcomes in Older Patients With Hip Fractures After Surgery: Systematic Review and Meta-Analysis

**DOI:** 10.2196/49482

**Published:** 2024-06-12

**Authors:** Suphawita Pliannuom, Kanokporn Pinyopornpanish, Nida Buawangpong, Nutchar Wiwatkunupakarn, Poppy Alice Carson Mallinson, Wichuda Jiraporncharoen, Chaisiri Angkurawaranon

**Affiliations:** 1 Department of Family Medicine Faculty of Medicine Chiang Mai University Chiang Mai Thailand; 2 Global Health and Chronic Conditions Research Group Chiang Mai University Chiang Mai Thailand; 3 Faculty of Epidemiology and Population Health London School of Hygiene & Tropical Medicine London United Kingdom

**Keywords:** home-based intervention, digital health interventions, postoperative care, older adults, hip fracture

## Abstract

**Background:**

Digital health interventions (DHIs) have been used to improve postoperative functional ability in older patients with hip fractures. However, there is limited information on the characteristics of home-based DHIs, and controversy exists regarding their impact on functional outcomes in this population.

**Objective:**

This study aims to provide an overview of the characteristics and effects of home-based DHIs on functional outcomes in older patients with hip fractures after surgery.

**Methods:**

We conducted a systematic review and meta-analysis following PRISMA (Preferred Reporting Items for Systematic Reviews and Meta-Analyses) guidelines. Five electronic medical databases (PubMed, Embase, Cochrane, ProQuest, and CINAHL) were searched up until January 3, 2023. We included clinical trials or randomized controlled trials (RCTs) in English involving home-based DHIs for postoperative care among older patients with hip fractures. Excluded studies involved patients not hospitalized, not discharged to home, not directly using DHIs, or with inaccessible full text. The PROSPERO registration number is CRD42022370550. Two independent reviewers screened and extracted data (SP and NB). Disagreements were resolved through discussion and agreement with the third author (KP). Home-based DHIs were characterized in terms of purpose and content, mode of delivery, and health care provider. Functional outcomes assessed included Timed Up and Go (TUG) test, Short Physical Performance Battery (SPPB), and Functional Independence Measure (FIM). Summary measures were calculated using mean differences with 95% CIs. Risk of bias was assessed using the Risk-of-Bias 2 assessment tool for RCTs and ROBINS-I for non-RCTs. The quality of evidence was assessed using GRADE (Grading of Recommendations Assessment, Development and Evaluation).

**Results:**

Of 2125 identified studies, 16 were included in the systematic review, involving 1467 participants. Six studies were included in the meta-analysis (4 for TUG, 4 for SPPB, and 2 for FIM). Home-based DHIs predominantly involved communication and feedback, education, and telerehabilitation. Telephone calls were the most common mode of delivery, followed by web-based software and mobile apps. Physical therapists were the main health care providers. The meta-analysis showed that home-based DHIs improved functional outcomes compared with usual care, with decreased TUG scores (mean difference=–7.89; 95% CI –10.34 to –5.45; *P*<.001), significantly increased SPPB scores (mean difference=1.11; 95% CI 0.51-1.72; *P*<.001), and increased FIM scores (mean difference=7.98; 95% CI 5.73-10.24; *P*<.001).

**Conclusions:**

Home-based DHIs that integrate communication and feedback, education, and telerehabilitation have demonstrated effectiveness in enhancing functional outcomes among older patients recovering from hip fractures after surgery. These interventions are commonly administered by physical therapists, who play a crucial role in facilitating and guiding the rehabilitation process. However, while the existing evidence supports the efficacy of such interventions, further research is needed to enhance our understanding and optimize the implementation of home-based DHIs for this specific population.

## Introduction

Hip fractures are a common and serious health issue [1-3], ranking among the top 10 causes of disability among older individuals [4]. The global prevalence is increasing, particularly among Asian people, who have the highest rate of hip fractures [4]. The mortality rate after they receive aggressive management in both surgery and rehabilitation is high, 10% in the first month [2,4] and 12%-36% in the first year [4,5]. Moreover, they have many consequences including functional decline [1], fear of falling [6], and reduced quality of life [2,4,5], and can lead to caregiver burden [1].

Apart from early surgery that can reduce mortality [7], the postoperative or transitional care after hospital discharge to home is also a crucial phase in the care process for patients with hip fracture [8]. The goal of treatment in this population is to improve functional outcomes, enhance independence, and reduce the risk of recurrent falls following surgery. Several home-based interventions, such as rehabilitation [5,9], dietary supplementation to prevent calorie and protein malnutrition [5], home hazard modification [5], fall prevention guidance [5,10], and timely and appropriate patient follow-up (including telephone calls and home visits) [5], as well as patient, family, and caregiver education and support [11], have been found beneficial in postoperative care among older patients with hip fractures. These interventions play a crucial role in improving patient functional ability, enhancing quality of life, reducing unplanned readmissions, minimizing complications and disabilities, and decreasing mortality [12].

Previous research found that combining postsurgical interventions for patients with fragility hip fractures across inpatient and outpatient settings may be able to improve physical function recovery and discovered that 63% of the studies were related to rehabilitation or medication or nutrition supplementation [13].

Over the past couple of decades, advancements in information and communication technologies have brought medical services to virtually all corners of the world [14]. Digital health interventions (DHIs) or the use of digital technology for health [15] has become a field of practice for using routine and innovative forms of information and communication technologies to address health needs [15]. DHIs have the potential to help address problems such as distance and access [15] and have been incrementally changing. DHIs do not only assist health care providers and patients receiving treatment but also benefit perfectly healthy people by providing health assessment [14] and health promotion. A recent review of home-based exercise programs delivered through DHIs for community-dwelling adults older than 65 years, involving a diverse study population with various health conditions, demonstrated improved physical function, particularly in terms of lower extremity strength [16]. Furthermore, many DHIs are specifically designed to detect, monitor, and provide care for specific health conditions [17], aiming to enhance various health aspects at home, even among older patients with hip fractures [18,19]. Previous studies have explored various types of home-based DHIs [18,19] with a focus on different interventions, such as rehabilitation, nutrition, and fall prevention, as well as different outcomes, including functional improvements, adherence to protocols, and the incidence of recurrent falls [9,19,20]. Typically, these previous studies have provided interventions to improve functional outcomes, and many of them have achieved success [16,19,21,22].

The scoping review elucidates the characteristics of DHIs for older adults who have undergone hip fracture surgery, with most studies focusing on physical therapy [23]. These DHIs were typically implemented in acute hospital settings and rehabilitation centers. However, there is a notable lack of information regarding DHIs specifically designed to improve functional outcomes in this population during transitional care settings. As a result, information on the characteristics of home-based DHIs in postoperative care among older patients with hip fractures is currently sparse, and there is a lack of information about specific home-based DHIs for this population [24]. Moreover, there is controversy over the conclusion that home-based DHIs have an impact on functional outcomes. Our systematic review and meta-analysis aimed to provide a general overview of the characteristics and effects of home-based DHIs in postoperative care on functional outcomes among older patients with hip fractures. This will lead to the development of home-based DHIs that can provide care for this population.

## Methods

### Ethical Considerations

We conducted a systematic review and meta-analysis and then reported according to the PRISMA (Preferred Reporting Items for Systematic Reviews and Meta-Analyses) 2020 guidelines (Multimedia Appendix 1) [25]. This study was approved by the Chiang Mai University Ethics Committee (FAM-2565-09330) and we registered our protocol in PROSPERO (CRD42022370550).

### Recruitment

#### Review Eligibility Criteria

We included the studies that related to (1) older adults (including patients aged 60 years or older) with hip fractures defined as any type of hip fracture, such as femoral neck, intertrochanteric, subtrochanteric fracture, and nonspecified type of hip fracture; (2) postoperative care (including transitional care, postdischarge care, and subacute care); and (3) home-based DHIs. In addition, the included studies that had (4) study design limited to clinical trials or randomized controlled trials (RCTs) and (5) compared between home-based DHIs (intervention) and usual care (comparator) in the English language with no date restriction. The following studies were excluded: patients enrolled in the emergency department but not admitted to the hospital, patients discharged from the hospital to a setting other than home, studies that involved DHIs that did not directly intervene with patients (including database management), and studies with inaccessible full-text or insufficient data for evaluating the characteristics of DHIs or quantitative synthesis.

#### Search Strategies

This study was systematically searched in 5 electronic medical databases, that is, PubMed, Embase, Cochrane, ProQuest, and CINAHL, with no date restriction. We searched until January 3, 2023. We searched in 3 heading terms including “hip fracture” AND “digital health” AND “post-operative” (Table S1 in Multimedia Appendix 2).

#### Review Selection

Duplicates were removed automatically by the EndNote X9 program (Clarivate Analytics) and the Rayyan website, as well as manually by SP and NB. Then, 2 authors (SP and NW) independently reviewed and screened all studies in title and abstract for study eligibility and 2 reviewers (SP and NB) independently screened the full paper for study selection. Disagreements were resolved through discussion and agreement with the third author (KP).

#### Data Extraction

Two authors (SP and NB) independently extracted data from the full original studies using a standard data collection form. Disagreements were resolved through discussion and agreement with the third author (KP). The data extracted included (1) the first author, (2) publication year, (3) study design, (4) study location, (5) population (including the number of patients, age, and sex), (6) the characteristic of home-based DHIs, (7) control group, (8) functional outcomes, and (9) other outcomes.

#### Characteristics of Home-Based DHIs

The characteristics of home-based DHIs focused on three dimensions, which included (1) purpose and content, (2) mode of delivery, and (3) health care provider. Two authors categorized subgroups in each dimension according to the definition (Table S2 in Multimedia Appendix 2) [17,26,27]. The categorizations possibly reported more than 1 category in each dimension. Purpose and content were categorized into 5 categories including education, telerehabilitation, communication and feedback, behavioral and lifestyle interventions or health coaching, and remote monitoring [17,26,27]. Modes of delivery were categorized into 4 categories including web-based software, telephone call, mobile apps, and sensor-based technology [17,26,27]. Health care providers were categorized into 6 categories including occupational therapists (OTs), physical therapists or physiotherapists (PTs), physicians, nurses, dieticians, and multidisciplinary teams. If the results were unable to be meta-analyzed, we conducted a narrative synthesis to provide an overview of the characteristics and effects of home-based DHIs on functional outcomes in this population.

#### Outcomes

In the meta-analysis, the studies that reported functional outcomes including Timed Up and Go (TUG) test, Short Physical Performance Battery (SPPB), and Functional Independence Measure (FIM) were considered. TUG is a tool that assesses functional walking ability and predicts fall risk in older adults. The shorter the time taken means better the balance function and walking ability, and the lower the risk of fall [28,29]. SPPB is a comprehensive assessment index that is used to evaluate the motor ability of the older adults and to predict the risk of falls. It consists of 3 parts that measure balance test, gait speed test, and 5 sit-to-stand tests, which assess static and dynamic balance, lower limb strength, and gait speed. The SPPB score runs from 0 to 12 and the higher the score mean the better the overall somatic ability and the lower the risk of fall [28]. FIM is a postdischarge follow-up evaluation test that consists of 18 items that measure patients’ somatic, verbal, social, and cognitive abilities. The score ranges from 18 to 126. Higher scores mean higher levels of functional independence and lower levels of dependence [28].

### Data Analysis

In meta-analysis, the outcomes included functional outcomes (such as TUG, SPPB, and FIM) reported as mean and SD at the end of the intervention for both the intervention group and the control group. Because the same methodology and range of outcomes were used in all studies to analyze the outcomes, the summary measure for meta-analysis was the mean difference with 95% CIs. Pooled-effect estimates of each functional outcome of interest were calculated by the random-effects DerSimonian-Laird model. Statistical heterogeneity among included studies was assessed using Cochran Q test and the percentage of total variability across studies due to heterogeneity (*I*^2^=0%-40%, low; *I*^2^=30%-60%, moderate; *I*^2^=50%-90%, substantial, and *I*^2^=75%-100%, considerable) [30]. Results of the meta-analysis and forest plot were performed using a standard software package (Stata, version 16.0; StataCorp LLC). *P* value of <.05 was considered statistically significant.

### Risks of Bias

We assessed the quality of each included study using the Risk of Bias 2 assessment tool (RoB 2; The Cochrane Collaboration) for the 14 RCT studies. In addition, 1 non-RCT and 1 quasi-experimental study were evaluated using the ROBINS-I (The Cochrane Collaboration) [31]. The results were visualized using robvis, a visualization tool [32]. This assessment was conducted independently by 2 authors, SP and NB, with any disagreements resolved through discussion and agreement with coauthor KP.

#### Grading the Quality of Evidence

We graded the quality of evidence using GRADE (Grading of Recommendations Assessment, Development and Evaluation) method [33]. This assessment was conducted independently by 2 authors, SP and NB, with any disagreements resolved through discussion and agreement with the third author, KP. The quality of evidence was categorized into 4 levels: very low, low, moderate, or high.

## Results

**Figure 1:**
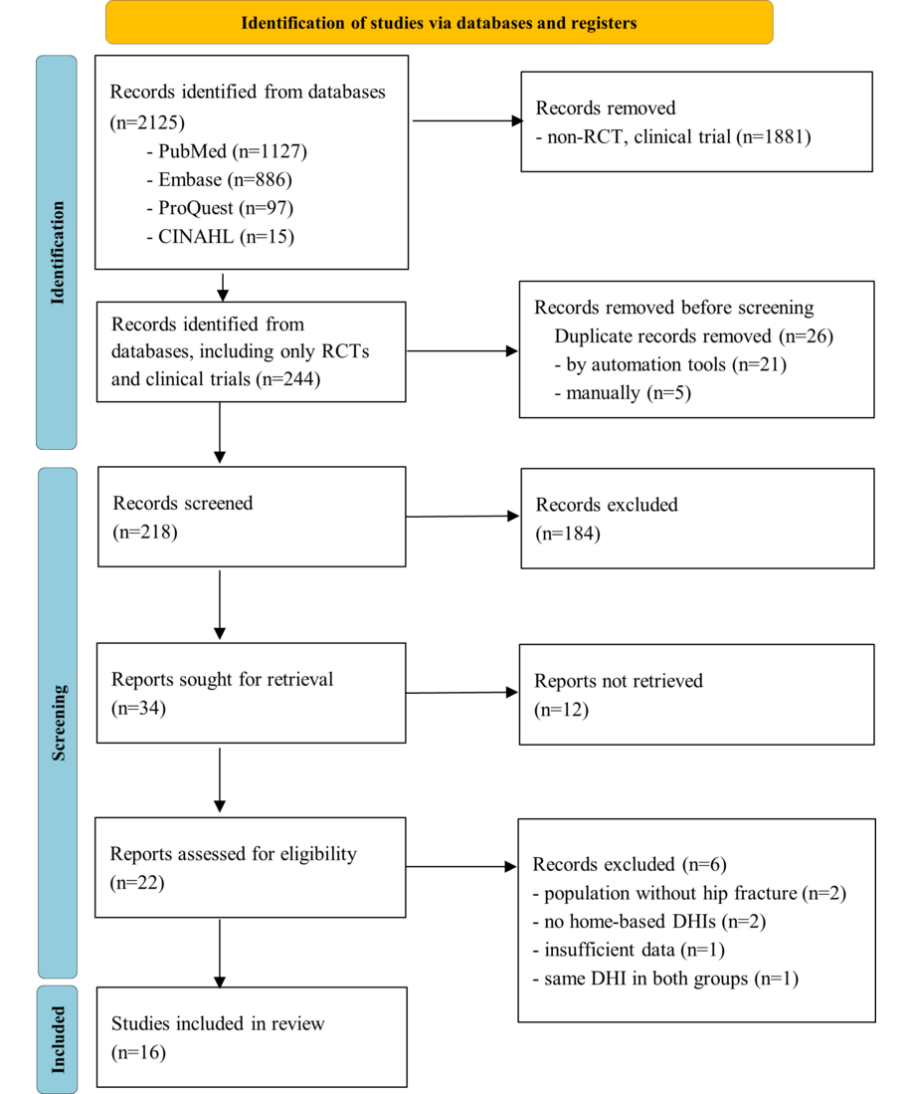
PRISMA (Preferred Reporting Items for Systematic Reviews and Meta-Analyses) 2020 flow diagram. DHI: digital health intervention; RCT: randomized controlled trial.

During the initial literature search, a total of 2125 studies were identified. Among them, 244 studies were clinical trials or RCTs. After screening the titles and abstracts for eligibility criteria, 218 studies were retrieved and further assessed. Subsequently, 22 studies underwent full-text screening. The reasons for excluding studies were as follows: 2 studies involved a population without hip fractures [34,35], 2 study did not include home-based DHIs [36,37], 1 study had insufficient data [38], and 1 study used the same DHIs in both groups [39]. Finally, a total of 16 studies were included in the systematic reviews (Figure 1), with a subset of 6 studies included in the meta-analysis (4 studies for TUG, 4 studies for SPPB, and 2 studies for FIM).

The characteristics of 16 studies are shown in Table 1 and Table S3 in Multimedia Appendix 2 [28,40-51]; 13 studies were 2-arm RCTs (compared DHIs with usual care), 1 study was 3-arm RCT (compared with DHIs add on cognitive behavioral therapy [CBT], only CBT, and usual care) [19], 1 study was non-RCT [52], and 1 study was quasi-experimental study [53], published between 2005 and 2022 (Figure S1 in Multimedia Appendix 2). Studies were conducted in Europe (n=7), America (n=5), and Asia (n=4). The total number of participants in the included studies was 1467.

**Figure 2:**
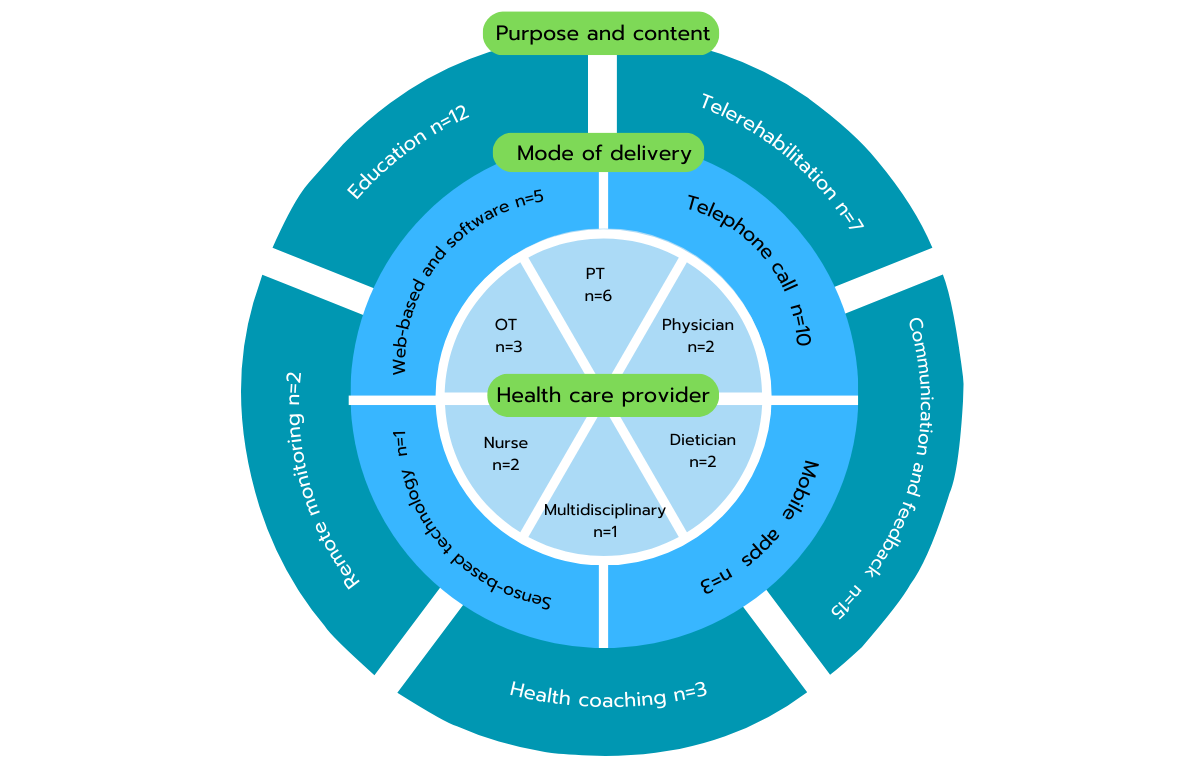
The characteristics of home-based digital health interventions in 3 dimensions. Multidisciplinary team: 1 study (sport science, PT, OT, and orthopedic consultation). The categorizations possibly reported more than 1 category in each dimension. OT: occupational therapist; PT: physical therapist or physiotherapist.

Figure 2 presents the characteristics of home-based DHIs. In terms of the purpose and content of home-based DHIs, most of the included studies (14 of 16) used multiple categories within each study. Two studies focused solely on communication and feedback. The common categories identified in this dimension were communication and feedback (n=15), education (n=12), telerehabilitation (n=7), health coaching (n=3), and remote monitoring (n=2). Regarding the mode of delivery, the majority of studies (13 of 16) used a single mode of delivery, while 3 studies used combined modes. The reported modes of delivery included telephone calls (n=10), web-based software platforms (n=5), mobile apps (n=3), and sensor-based technologies (n=1). The involvement of health care providers in DHIs varied. PTs were involved in 6 studies, OTs in 3 studies, physicians in 2 studies, nurses in 2 studies, dieticians in 2 studies, and a multidisciplinary team in 1 study.

The majority of research indicates that home-based DHIs have shown a beneficial effect on mobility and physical function, as evidenced by improvements in scores on tests such as TUG, SPPB, and FIM [28,40-43,52]. Furthermore, these studies suggest that home-based DHIs are feasible for enhancing various aspects of hip fractures after surgery, such as improving physical function (daily activities and instrumental activities of daily living) [19,44,53], improved adherence for self-care [45], enhancing nutritional intake and status [46,47], and potentially preventing urinary incontinence and reducing its frequency [48] (Table 1).

**Figure 3:**
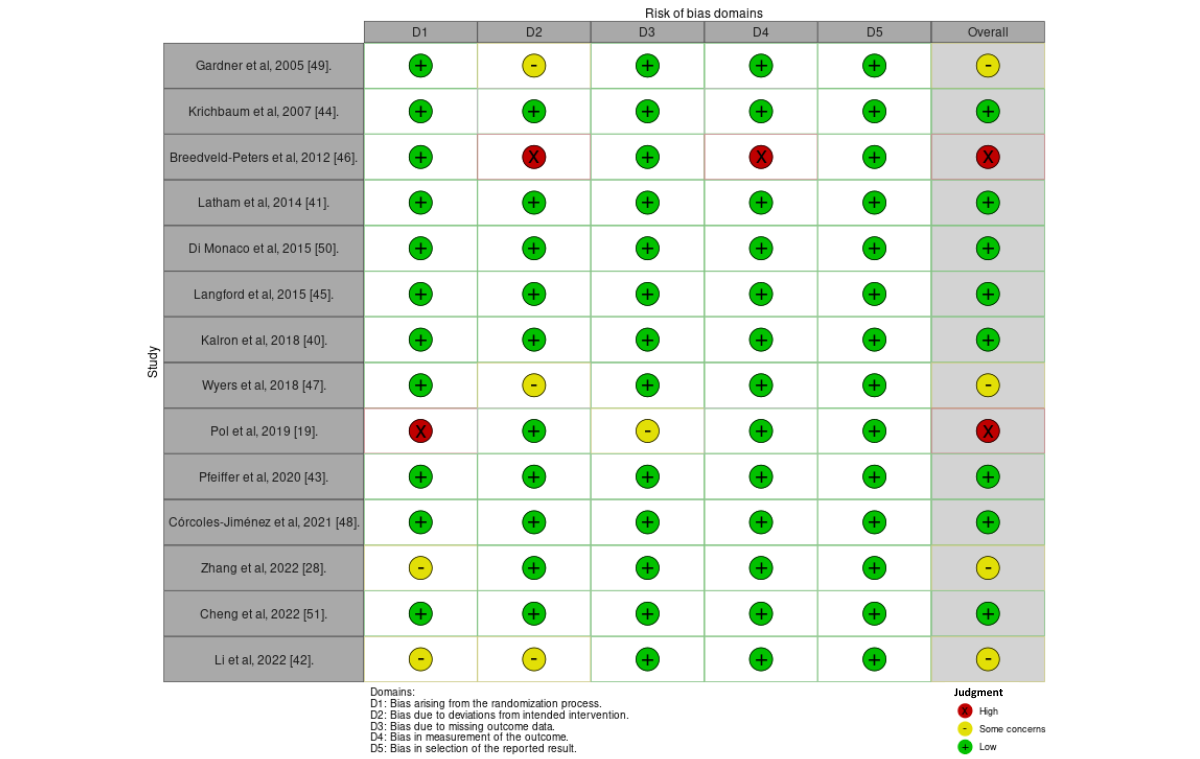
Risk of bias of included studies (14 randomized controlled trials).

**Figure 4:**
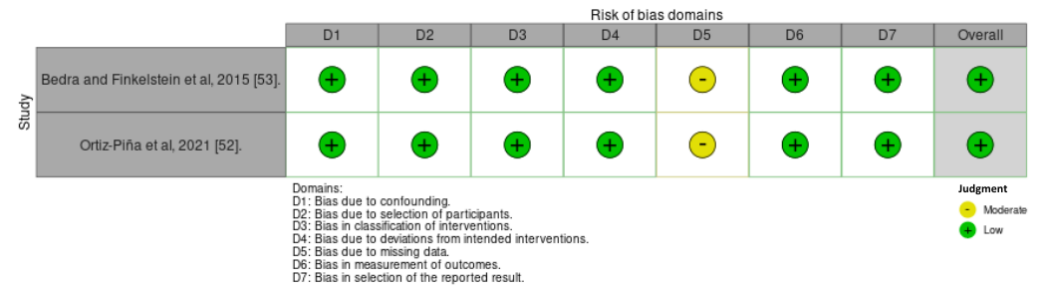
Risk of bias of included studies (2 non–randomized controlled trials).

**Figure 5:**
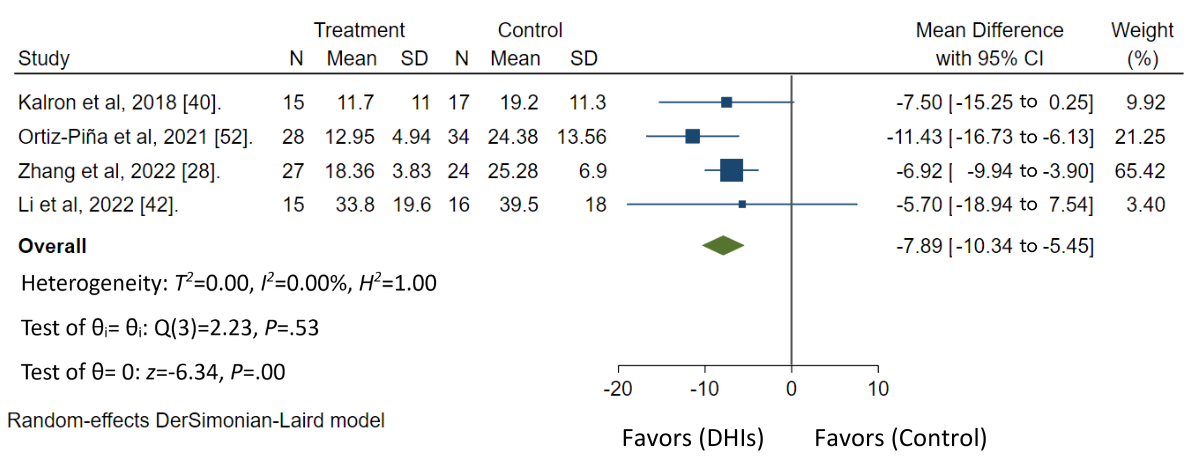
Summary measure Timed Up and Go for meta-analysis [28,40,42,52]. DHI: digital health intervention.

**Figure 6:**
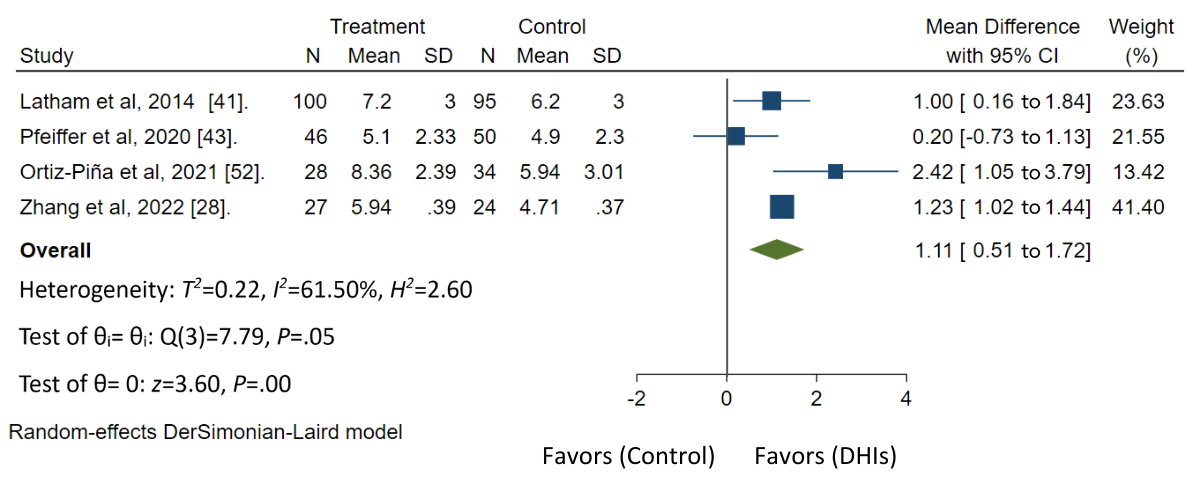
Summary measure Short Physical Performance Battery for meta-analysis [28,41,43,52]. DHI: digital health intervention.

**Figure 7:**
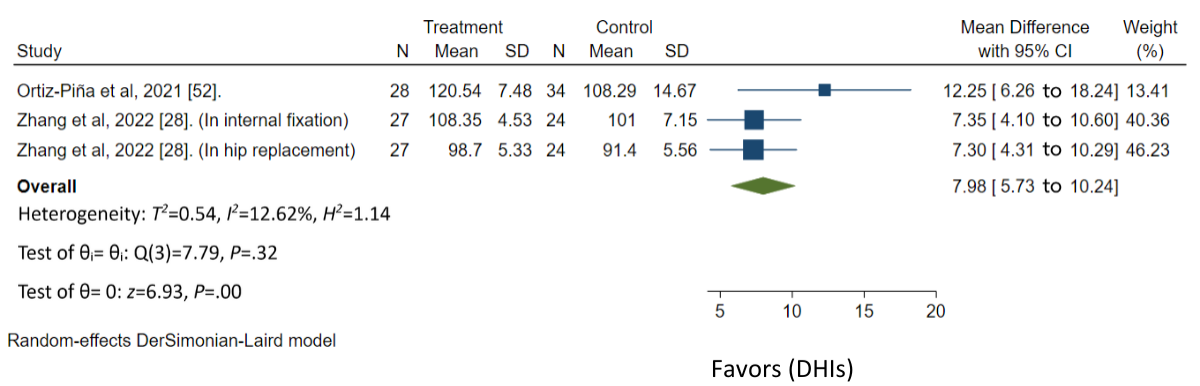
Summary measure Functional Independence Measure for meta-analysis [28,52]. DHI: digital health intervention.

Ten of 16 studies had low risk of bias, 4 studies had some concerns, and 2 studies had high risk of bias (Figure 3 [19,28,40-45,47-51] and Figure 4 [52,53]). In the GRADE assessment of the quality of evidence across all outcomes, findings revealed moderate quality of evidence for TUG based on 4 studies, SPPB based on 4 studies, and for FIM based on 2 studies. (Table S4 in Multimedia Appendix 2). The forest plot analysis demonstrated that home-based DHIs resulted in a significant decrease in the TUG score (mean difference=–7.89; 95% CI –10.34 to –5.45; *P*<.001), improvement in SPPB score (mean difference=1.11; 95% CI 0.51-1.72; *P*<.001), and improvement in FIM score (mean difference=7.98; 95% CI 5.73-10.24; *P*<.001). Heterogeneity was low in both TUG (*I*^2^=0.00%; *P*<.53) and FIM scores (*I*^2^=12.62%; *P*<.32). However, substantial heterogeneity was observed in SPPB scores (*I*^2^=61.50%; *P*<.05). These findings are shown in Figures 5-7, respectively.

**Table 1:** Characteristics of the included studies.

First author, year of publication	Population	Intervention	Purpose and content of DHIs^a^	Mode of delivery	Health care provider	Control	Functional outcomes	Conclusion
Gardner et al (2005) [49]	80: femoral or intertrochanteric fracture, mean age 82 years, female 78% I^b^: 40 (end 36) C^c^: 40 (end 36)	Educational intervention with 5 discussion questions regarding osteoporosis treatment Single telephone call to remind questions during a follow-up	Education, communication, and feedback	Telephone call	Primary medical physician	Brochure (methods for preventing falls)	N/A^d^	Patients provided vs not provided with information and questions were more likely to receive appropriate intervention
Krichbaum (2007) [44]	33: hip fracture average age 78.87 years (range 72-85 years), female 73%	Nursing postacute care coordination intervention First month: face-to-face then face-to-face or telephone until 6 months Health assessment	Education, communication, and feedback	Telephone call	Gerontologic advanced practice nurse	Usual care	Intervention led to significantly improved function in most ADLs^e^ and IADLs^f^ measured using the functional status index score	I group had better function at 12 months on several activities and IADLs
Breedveld-Peters et al (2012) [46]	66: hip fracture, mean age 76 years (range 55-92 years), female 74%	Dietetic counseling (telephone call 5 visits and face-to-face 5 visits) and ONS^g^ for 3 months	Communication and feedback	Telephone call	Dieticians	Usual nutritional care	N/A	Face-to-face and telephone call nutritional counseling are feasible
Latham et al (2014) [41]	232: hip fracture, mean age 78 (SD 9.9) years, female 69% I: 120 (end 100) C: 112 (end 95)	Functionally oriented exercises, home exercise program 3 times per week for 6 months Monthly telephone call DVD version of the program	Education, telerehabilitation, communication and feedback, and health coaching	Telephone call and web-based software	PT^h^	In-home and telephone-based cardiovascular nutrition education	I-group significantly improved in SPPB^i^ between group difference 0.8 (95% CI 0.4-1.2; *P*<.01)	Home-based functionally oriented exercise program resulted in modest improvement in physical function at 6 months
Di Monaco et al (2015) [50]	169: hip fracture, mean age 78.7 (SD 7.2) years in I-group, C-group 79.3 (8.0) years I: 84 (end 78) C: 85 (end 75)	Multidisciplinary rehabilitation program during hospitalization Single telephone call to reinforce the targeted recommendations for fall prevention	Education, communication, and feedback	Telephone call	OT^j^	Multidisciplinary rehabilitation program during hospitalization	At least 1 fall during follow-up; relative risk 1.06 (95% CI 0.48-2.34)	This study did not support postdischarge single telephone call to reinforce the recommendations for fall prevention
Bedra and Finkelstein (2015) [53]	10: hip fracture, mean age 77 (SD 9) years, female 60% Preintervention: 10 Postintervention: 10	Postcomprehensive telerehabilitation system to support individualized exercise program using home automated telemanagement tailored feedback multimedia education individualized to patient’s need	Education, telerehabilitation, communication, and feedback	Web-based software	PT	Preintervention	*P* value pre- and postcomparing Modified Barthel Index improved (*P*=.10) LEFS^k^ significantly improved (*P*=.03)	Home-based telerehabilitation may be a viable model for postacute hip fracture recovery
Langford et al (2015) [45]	30: hip fracture, mean age 81.5 (range 61-97) years, female 63.33% I: 15 (end 11) C: 15 (end 15)	Educational (1 hour in-hospital session) plus 5 postdischarge telephone call coaching	Education, health coaching, communication and feedback	Telephone call	PT	Usual care plus 1 hour in-hospital educational session	No differences between groups for grip strength, gait speed	Telephone coaching for older adults after hip fracture to improve adherence to mobility recovery goals has feasibility
Kalron et al (2018) [40]	32: femoral neck fracture, mean age 66.2 (SD 11.6) years, female 62.5% I: 20 (end 15) C: 20 (end 17)	Home-based telerehabilitation: video clips of common rehabilitation exercises focusing on the lower limbs, 40-50 minute/session	Education, tele-rehabilitation, communication, and feedback	Web-based software	PT (specialized in neurological rehabilitation)	Home-based exercise booklet	I-group greater improved significantly in 5/6 tests compare with C-group: TUG, 2-minute walk, sit to stand test, Walking speed, Mean step length	Telerehabilitation generated a positive effect on mobility in people following hip surgery
Wyers et al (2018) [47]	152: hip fracture, mean age 77 (SEM 1.2) years in I-group, C-group 76 (SEM 1.1) years, female 71% I: 73 (end: 63) C: 79 (end: 68)	Intensive nutritional intervention 10 counseling: 2 sessions in hospitalization and 8 sessions of weekly dietetic consultation (3 face-to-face, 5 telephone calls) Energy-protein–enriched diet and ONS for 3 months	Communication and feedback	Telephone call	Dietician	Usual nutritional care	Does not affect functional and any outcome	Intensive nutritional intervention after hip fracture improved nutritional intake and status but not LOS^l^ or clinical outcomes
Pol et al (2019) [19]	240: hip fracture, mean age 83.8 (SD 6.9) years, female 80%(3 arms) CBT^m^ on OT with sensor: 76 CBT on OT: 87 Usual 77	CBT-based occupational therapy (weekly session coaching of skilled nursing facility, 4 home visits, 4 telephone consultation) Sensor monitoring (physical activity monitor, motion sensors)	Remote monitoring, health coaching, communication and feedback	Sensor-based technology, telephone call	OT	Usual care CBT-based occupational therapy	Mean patient-reported daily functioning in the CBT-based OT with sensor monitoring was larger than usual group (difference 1.17 (95% CI 0.47-1.87; *P*=.001).	Rehabilitation program of sensor-monitoring-informed OT more effective in improving patient-reported daily functioning at 6 months than usual care
Pfeiffer et al (2020) [43]	115: hip and pelvic fracture, mean age 82.5 (SD 6.8) years, female 76% I: 57 (end 46) C: 58 (end 50)	8 individual sessions during inpatient rehabilitation Additional support via 1 home visit, 4 telephone calls	Communication and feedback, education	Telephone call	PT or sports therapist	Geriatric inpatient rehabilitation	End point SPPB improved in favor of I-group from baseline to follow-up (2.03 vs 0.70; *P*=.002, *d*=0.58)	Intervention improved psychological and physical performance measures but did not increase daily walking duration
Ortiz-Piña et al (2021) [52]	71: hip fracture, mean age 75.86 (SD 5.79) years in I-group and 80.38 (SD 5.54) years in C-group, female 75.8% I: 35 (end 28) C: 36 (end 34)	@ctivehip: multidisciplinary telerehabilitation program supervised by family caregivers, 50-60 minutes per session, 5 sessions per week in 2 we-based components: 3 exercise sessions + 2 OT sessions Videoconference (if need)	Education, telerehabilitation, communication, and feedback	Web-based software	Multidisciplinary (sport sciences professionals, PT, OT, and orthopedic surgeon consultants)	Home-based usual outpatient rehabilitation	I-group higher FIM^n^ (high effect size), better performance in TUG^o^ (medium effect size) significantly compared with C-group No statistically significant differences between group postintervention in SPPB	Telerehabilitation had better results in functional independence and physical condition (self-report and performance-based) than usual care
Córcoles-Jiménez et al, (2021) [48]	109: hip fracture, mean age 80.56 (SD 6.65) years, female 70.6% I: 53 (end 51) C: 56 (end 51)	Urinary habit training (education): in-hospital stay Telephonic reinforcement to remind the recommended activities, repeating list of activities	Communication and feedback, education	Telephone call	Nurse	Usual medical care	Mean of independence in ADL I-group 83.03 (SD 23.41), C-group 71.37 (SD 26.83) (*P*=.005)	Educational and remind system prevents the development of UI^p^ and decreases the number of episodes
Zhang et al (2022) [28]	58: hip fracture, mean age 77.0 (SD 7.89) years in I-group, C-group 75.17 (SD 7.73) years, female 64.7% I: 29 (end 27) C: 29 (end 24)	Home-based telerehabilitation. Personalized rehabilitation programs, video, health knowledge Remote-monitoring vital sign, assessment and guidance Appointment for consultation	Education, telerehabilitation, communication and feedback, and remote monitoring	Mobile apps (patient sided), web-based software (physician side)	Physician	Former telephone follow-up at 2 weeks and 1, 2, 3, and months after discharge	The Harris hip score, FIM of I-group higher than C-group at 1 and 3 months (*P*<.05) TUG, SPPB I-group better than C-group at 3 months (*P*<.05)	Home-based telerehabilitation improves functional recovery of hip joint and enhances ability to perform ADL and somatic integration to a certain extent
Cheng et al (2022) [51]	39: hip fracture, mean age 77.4 years, female 49.7% I: 19 C: 20	Mobile apps in delivering home-based rehabilitation program. Briefing session before discharge Perform exercise along exercise video in app	Education, telerehabilitation, communication, and feedback	Mobile apps	PT	Home-based rehabilitation program using an exercise pamphlet	No difference in modified functional ambulatory category, elderly mobility scale, LEFS at 1 and 2 months	Mobile apps improved exercise adherence but did not improve physical performance, self-efficacy, and reduced exercise adherence
Li et al (2022) [42]	31: hip fracture, mean age 76.5 (SD 8.6) years in I-group, 82.1 (SD 9.7) years in C-group, female 80.6% I: 15 C: 16	Home program using the Caspar Health e-system. Tailor-made telerehabilitation program through e-system calendar Video, pictures, and written and verbal instructions shown on app Patient feedback and OT update program	Education, telerehabilitation, communication and feedback	Mobile apps	OT	Home program paper sheets and log sheets	I-group significant improvement in IADL (*P*=.010) at postintervention and follow-up No significant differences in TUG, functional reach test, and modified Barthel index	Telerehabilitation via smartphone as alternative home program for use in OT practice with older after hip fracture surgery

^a^DHI: digital health intervention.

^b^I, I-group: intervention group.

^c^C, C-group: control group.

^d^N/A: not applicable.

^e^ADLs: activities of daily living.

^f^IADLs: instrument activities of daily living.

^g^ONS: oral nutritional supplement.

^h^PT: physical therapist or physiotherapist.

^i^SPPB: Short Physical Performance Battery.

^j^OT: occupational therapist.

^k^LEFS: Lower Extremity Functional Scale.

^l^LOS: length of stay.

^m^CBT: cognitive behavioral therapy.

^n^FIM: Functional Independence Measure.

^o^TUG: Timed Up and Go.

^p^UI: urinary incontinent.

## Discussion

### Principal Findings

In this systematic review, we described the characteristics of home-based DHIs in postoperative care for older patients with hip fractures. Our findings revealed that the majority of home-based DHIs encompassed multiple components in terms of purpose and content. Furthermore, we observed that these interventions were typically delivered through a single mode of delivery and were provided by health care professionals. Importantly, our meta-analysis demonstrated that home-based DHIs were effective in improving functional outcomes for older patients during the postoperative phase of care for hip fractures.

Home-based DHIs in older patients with hip fractures after surgery were first described in 2005. This upward trend can be attributed to the use of DHIs in clinical care for various conditions, including frailty [17], diabetes mellitus [54], and chronic disease [55]. We found that the frequency of research on DHIs for older individuals with hip fractures increased due to the aging population on the continent and the development of DHIs following the COVID-19 pandemic [23]. Subsequently, policy briefs for DHIs were adopted and implemented, incorporating a highly developed health care infrastructure with advanced information technology systems, financial support, and health care provider training [56]. Looking ahead, it is predicted that the role of DHIs will continue to expand in line with the global strategy for digital health 2020-2024 [57] and the WHO guideline recommendations on digital interventions for health system strengthening in 2018 [15].

The majority of studies used multicomponent approaches, considering the purpose and content of home-based DHIs in postoperative care for older individuals with hip fractures. Freitag and Magaziner [24] recommended that postoperative care in this population may require multiple interventions provided individually or through coordinated management. In this study, the most common categories of purpose and content were communication and feedback, combined with education in areas such as rehabilitation [40-42,51-53], fall prevention [50], and osteoporosis [49]. A lack of knowledge and awareness was identified as an important barrier for self-care [58]. During the postoperative period, patients with hip fracture and their caregivers receive numerous recommendations, including early ambulation, exercise and rehabilitation programs, preventing postoperative complications, modifying home hazards, and taking nutrition supplements [24]. Therefore, effective communication skills with specific feedback are crucial for delivering accurate knowledge and information and ultimately leading to positive functional outcomes [24,59,60].

Telerehabilitation was the third most common content of home-based DHIs. It is essential for every patient with a hip fracture to receive rehabilitation in some setting, including outpatient rehabilitation, nursing homes, and home care. Rehabilitation is one of the most crucial aspects of postoperative care, as it contributes to recovering functional outcomes, patient ability, and overall quality of life [24]. Crotty et al [61] found that a home-based rehabilitation program yielded similar outcomes and placed less burden on caregivers compared with a hospital program. Following the COVID-19 pandemic, telerehabilitation has gained even greater significance in clinical care for patients with postoperative hip fracture. According to Seron et al [22], the most common interventions in telerehabilitation include therapeutic exercises, functional training, and education. Therefore, telerehabilitation is a vital option for rehabilitation as it reduces hospital visits, decreases the cost of hospital visits, and still enhances functional outcomes.

The telephone call was the most common mode of delivery in this study and previous studies due to its ease of use, availability in most households, and effectiveness in communicating and providing simple educational information to patients and their families [24,60]. This is especially true in the Thai context, where a high number of older adults do not use the internet in their everyday lives. Therefore, using telephone calls should enhance the chance for accessing care with DHIs. Multiple episodes of telephone calls may increase adherence and functional outcomes more than a single telephone call [43,50]. However, based on our findings, using the telephone alone was less likely to achieve the goal of improving functional outcomes than other modes of delivery for patients with postoperative hip fracture. The limitations of telephone calls include only voice evaluation and advice and the absence of video connection. Telephone calls are limited when health care providers need to assess patients and provide exercise programs through video calls [62]. Therefore, telerehabilitation should use web-based software or mobile apps as the mode of delivery. Both platforms can provide real-time or on-demand video calls and chat, allowing for the delivery of rehabilitation programs, tracking of progress, and modification of the program based on the patient’s status [24].

Most studies used only 1 health care provider who played an important role in this intervention. The top 2 providers were PT and OT, who addressed the population's rehabilitation needs during the recovery phase. They provided instructional materials and self-management guidance at home, leading to improvements in functional abilities, as shown by the results. However, a program that included only dieticians did not have evidence of improving the patients' status. This detail could be an insightful consideration for providers when developing care programs. While nutritional improvement is required following orthopedic surgery [63], focusing solely on nutrition may not be sufficient for patients to recover from their postoperative condition; a combination of nutrition and physical therapy interventions can maximize function [64]. Therefore, to provide home-based DHIs, health care providers should allow patients and families access to a specific scope of care that includes a rehabilitation program. In addition, given the complexity of hip fracture patients, a multidisciplinary approach is essential, typically involving early geriatric assessment [24]. This approach can enhance functional outcomes, reduce hospital stays, and decrease the future need for institutional care. Although the evidence regarding the effectiveness of multidisciplinary teams in this population is unclear [23], holistic care, such as holistic assessment, rehabilitation, and nutrition, remains important.

In the meta-analysis, it was found that home-based DHIs have a positive impact on enhancing functional outcomes by improving scores on tests such as TUG, SPPB, and FIM even after the interventions end [19,28,40-53]. The differences in population caused variances in the mean difference of TUG reduction. Although this study demonstrated that home-based DHIs have a positive impact on enhancing functional outcomes, further studies may be needed across other functional outcomes to provide a comprehensive summary of the results. The characteristics of home-based DHIs that were associated with increased functional outcomes included a combination of telerehabilitation, effective communication and feedback, and educational components. The modes of delivery commonly used were web-based software, and mobile apps, similar to previous literature [23]. Whether it involves a multidisciplinary team or a single health care provider, the inclusion of physical therapy content in home-based DHIs was found to improve functional outcomes. These findings highlight the importance of incorporating rehabilitation or physical therapy content through telehealth care. This may be particularly beneficial in the context of orthopedic surgery, where reduced limb usage can lead to muscle weakness and functional disability [65]. By enhancing movement through appropriate rehabilitation, improvements in range of motion, muscle strength, and overall performance can be achieved [65]. In addition, this study demonstrated that home-based DHIs potentially have a positive impact on improving physical function and activities of daily living, self-care, and nutritional status. Further studies may be needed to provide a comprehensive summary of these results. Future research should aim to include a larger number of studies reporting patient outcomes, ensure consistency in time points for outcome assessment, and explore the effectiveness of DHIs in diverse populations with hip fractures. For example, this could involve studying different underlying health conditions, various types of hip fractures or surgeries, or varying levels of functional impairment. Such research can help identify which subgroups benefit most from home-based DHIs. In addition, further research on cost-effectiveness of home-based DHIs compared with traditional interventions can inform health care decision makers and potentially lead to more widespread adoption of these approaches.

### Limitations

In this study, we demonstrated the common characteristics of home-based DHIs that have shown benefits in improving functional outcomes. This finding holds promise for the development of home-based DHIs aimed at enhancing functional outcomes. However, it is important to acknowledge the limitations encountered during the study. First, only a limited number of studies (6 in total) reported the functional outcomes of interest, which may restrict the generalizability of our findings. Second, although a summary analysis of functional results was conducted, the time points at which these outcomes were measured varied among the included studies. The duration of the intervention itself could potentially impact the observed differences in outcomes. Third, most studies did not specify the type of hip fracture and the type of surgery. Their populations were characterized as patients with hip fractures. The specific type of hip fracture was presented only in 2 studies. Consequently, we were unable to conduct a subgroup analysis**.** Moreover, it is important to note that our study exclusively focused on DHIs used in the care of postoperative populations with hip fractures. This narrow scope may limit the applicability of our findings to nonsurgical hip fracture populations, where the intervention strategies and approaches may differ. Furthermore, our meta-analysis mainly focused on functional outcomes, but there are other factors that impact patient overall outcomes, such as fear of falling, pain control, and caregiver burden, which have not been conclusively examined in this study. These limitations should be taken into consideration when interpreting the results.

### Conclusions

Overall, our findings suggest that home-based DHIs, particularly those incorporating telerehabilitation, offer a promising approach to enhance the recovery and functional abilities of older patients following hip fracture surgery. These interventions have the potential to improve the quality of care and patient outcomes in this population. Further research and implementation of home-based DHIs are warranted to fully leverage their benefits in postoperative care for older patients with hip fractures.

## Appendix

Multimedia Appendix 1 PRISMA (Preferred Reporting Items for Systematic Reviews and Meta-Analyses) checklist.

Multimedia Appendix 2 Searching and details of included articles.

## Abbreviations

CBT: cognitive behavioral therapy
DHI: digital health intervention
FIM: Functional Independence Measure
GRADE: Grading of Recommendations Assessment, Development, and Evaluation
OT: occupational therapist
PT: physical therapist or physiotherapist
RCT: randomized controlled trial
SPPB: Short Physical Performance Battery
TUG: Timed Up and Go

## Data Availability

All data generated or analyzed during this study are included in the tables and figures of this published paper.

## References

[1] Asif M, Cadel L, Kuluski K, Everall AC, Guilcher SJT (2020). Patient and caregiver experiences on care transitions for adults with a hip fracture: a scoping review. Disabil Rehabil.

[2] Parker M, Johansen A (2006). Hip fracture. BMJ.

[3] Veronese N, Maggi S (2018). Epidemiology and social costs of hip fracture. Injury.

[4] Bhandari M, Swiontkowski M (2017). Management of acute hip fracture. N Engl J Med.

[5] LeBlanc KE, Muncie HL, LeBlanc LL (2014). Hip fracture: diagnosis, treatment, and secondary prevention. Am Fam Physician.

[6] Rico CLV, Curcio CL (2022). Fear of falling and environmental factors: a scoping review. Ann Geriatr Med Res.

[7] Colais P, Di Martino M, Fusco D, Perucci CA, Davoli M (2015). The effect of early surgery after hip fracture on 1-year mortality. BMC Geriatr.

[8] Storm M, Siemsen IMD, Laugaland KA, Dyrstad DN, Aase K (2014). Quality in transitional care of the elderly: key challenges and relevant improvement measures. Int J Integr Care.

[9] Mahran DG, Farouk O, Ismail MA, Alaa MM, Eisa A, Ragab II (2019). Effectiveness of home based intervention program in reducing mortality of hip fracture patients: a non-randomized controlled trial. Arch Gerontol Geriatr.

[10] Fernandez MA, Griffin XL, Costa ML (2015). Management of hip fracture. Br Med Bull.

[11] (2016). Transitions of Care: Technical Series on Safer Primary Care. World Health Organization.

[12] Roper KL, Ballard J, Rankin W, Cardarelli R (2017). Systematic review of ambulatory transitional care management (TCM) visits on hospital 30-day readmission Rates. Am J Med Qual.

[13] Phang JK, Lim ZY, Yee WQ, Tan CYF, Kwan YH, Low LL (2023). Post-surgery interventions for hip fracture: a systematic review of randomized controlled trials. BMC Musculoskelet Disord.

[14] Fong B, Fong A, Li C (2020). Telemedicine Technologies: Information Technologies in Medicine and Digital Health.

[15] (2019). WHO Guideline: Recommendations on Digital Interventions for Health System Strengthening. World Health Organization.

[16] Solis-Navarro L, Gismero A, Fernández-Jané C, Torres-Castro R, Solá-Madurell M, Bergé C, Pérez LM, Ars J, Martín-Borràs C, Vilaró J, Sitjà-Rabert M (2022). Effectiveness of home-based exercise delivered by digital health in older adults: a systematic review and meta-analysis. Age Ageing.

[17] Linn N, Goetzinger C, Regnaux JP, Schmitz S, Dessenne C, Fagherazzi G, Aguayo GA (2021). Digital health interventions among people living with frailty: a scoping review. J Am Med Dir Assoc.

[18] Gilboa Y, Maeir T, Karni S, Eisenberg ME, Liebergall M, Schwartz I, Kaufman Y (2019). Effectiveness of a tele-rehabilitation intervention to improve performance and reduce morbidity for people post hip fracture—study protocol for a randomized controlled trial. BMC Geriatr.

[19] Pol MC, Riet GT, Hartingsveldt MV, Kröse B, Buurman BM (2019). Effectiveness of sensor monitoring in a rehabilitation programme for older patients after hip fracture: a three-arm stepped wedge randomised trial. Age Ageing.

[20] Montero-Odasso MM, Kamkar N, Pieruccini-Faria F, Osman A, Sarquis-Adamson Y, Close J, Hogan DB, Hunter SW, Kenny RA, Lipsitz LA, Lord SR, Madden KM, Petrovic M, Ryg J, Speechley M, Sultana M, Tan MP, van der Velde N, Verghese J, Masud T, Task Force on Global Guidelines for Falls in Older Adults (2021). Evaluation of clinical practice guidelines on fall prevention and management for older adults: a systematic review. JAMA Netw Open.

[21] Kuijlaars IAR, Sweerts L, Nijhuis-van der Sanden MWG, van Balen R, Staal JB, van Meeteren NLU, Hoogeboom TJ (2019). Effectiveness of supervised home-based exercise therapy compared to a control intervention on functions, activities, and participation in older patients after hip fracture: a systematic review and meta-analysis. Arch Phys Med Rehabil.

[22] Seron P, Oliveros MJ, Gutierrez-Arias R, Fuentes-Aspe R, Torres-Castro RC, Merino-Osorio C, Nahuelhual P, Inostroza J, Jalil Y, Solano R, Marzuca-Nassr GN, Aguilera-Eguía R, Lavados-Romo P, Soto-Rodríguez FJ, Sabelle C, Villarroel-Silva G, Gomolán P, Huaiquilaf S, Sanchez P (2021). Effectiveness of telerehabilitation in physical therapy: a rapid overview. Phys Ther.

[23] Zhang J, Yang M, Ge Y, Ivers R, Webster R, Tian M (2022). The role of digital health for post-surgery care of older patients with hip fracture: a scoping review. Int J Med Inform.

[24] Freitag MH, Magaziner J (2006). Post-operative considerations in hip fracture management. Curr Rheumatol Rep.

[25] Page MJ, McKenzie JE, Bossuyt PM, Boutron I, Hoffmann TC, Mulrow CD, Shamseer L, Tetzlaff JM, Akl EA, Brennan SE, Chou R, Glanville J, Grimshaw JM, Hróbjartsson A, Lalu MM, Li T, Loder EW, Mayo-Wilson E, McDonald S, McGuinness LA, Stewart LA, Thomas J, Tricco AC, Welch VA, Whiting P, Moher D (2021). The PRISMA 2020 statement: an updated guideline for reporting systematic reviews. BMJ.

[26] Gentili A, Failla G, Melnyk A, Puleo V, Tanna GLD, Ricciardi W, Cascini F (2022). The cost-effectiveness of digital health interventions: a systematic review of the literature. Front. Public Health.

[27] Gagnon MP, Sasseville M, Leblanc A (2022). Classification of digital mental health interventions: a rapid review and framework proposal. Stud Health Technol Inform.

[28] Zhang YY, Zhang YG, Li Z, Li SH, Xu WG (2022). Effect of home-based telerehabilitation on the postoperative rehabilitation outcome of hip fracture in the aging population. Orthop Surg.

[29] Barry E, Galvin R, Keogh C, Horgan F, Fahey T (2014). Is the Timed Up and Go test a useful predictor of risk of falls in community dwelling older adults: a systematic review and meta-analysis. BMC Geriatr.

[30] Deeks JJ, Higgins JP, Altman DG (2019). Analysing data and undertaking meta-analyses. Cochrane Collaboration.

[31] Sterne JA, Hernán MA, Reeves BC, Savović J, Berkman ND, Viswanathan M, Henry D, Altman DG, Ansari MT, Boutron I, Carpenter JR, Chan AW, Churchill R, Deeks JJ, Hróbjartsson A, Kirkham J, Jüni P, Loke YK, Pigott TD, Ramsay CR, Regidor D, Rothstein HR, Sandhu L, Santaguida PL, Schünemann HJ, Shea B, Shrier I, Tugwell P, Turner L, Valentine JC, Waddington H, Waters E, Wells GA, Whiting PF, Higgins JP (2016). ROBINS-I: a tool for assessing risk of bias in non-randomised studies of interventions. BMJ.

[32] McGuinness LA, Higgins JPT (2021). Risk-of-bias VISualization (robvis): an R package and Shiny web app for visualizing risk-of-bias assessments. Res Synth Methods.

[33] Schünemann H, Higgins J, Vist G, Glasziou P, Akl E, Skoetz N (2019). Completing ‘Summary of findings’ tables and grading the certainty of the evidence. Cochrane Handbook for Systematic Reviews of Interventions.

[34] Dallimore RK, Asinas-Tan ML, Chan D, Hussain S, Willett C, Zainuldin R (2017). A randomised, double-blinded clinical study on the efficacy of multimedia presentation using an iPad for patient education of postoperative hip surgery patients in a public hospital in Singapore. Singapore Med J.

[35] Crawford DA, Lombardi AV, Berend KR, Huddleston III JI, Peters CL, DeHaan A, Zimmerman EK, Duwelius PJ (2021). Early outcomes of primary total hip arthroplasty with use of a smartphone-based care platform: a prospective randomized controlled trial. Bone Joint J.

[36] Jagos H, Pils K, Haller M, Wassermann C, Chhatwal C, Rafolt D, Rattay F (2017). Mobile gait analysis via eSHOEs instrumented shoe insoles: a pilot study for validation against the gold standard GAITRite. J Med Eng Technol.

[37] Hou YJ, Zeng SH, Lin CC, Yang CT, Huang HL, Chen MC, Tsai HH, Liang J, Shyu YIL (2022). Smart clothes-assisted home-nursing care program for family caregivers of older persons with dementia and hip fracture: a mixed-methods study. BMC Geriatr.

[38] Nahm ES, Resnick B, DeGrezia M, Brotemarkle R (2009). Use of discussion boards in a theory-based health web site for older adults. Nurs Res.

[39] Lee H, Lee SH (2022). Effectiveness of multicomponent home-based rehabilitation in elderly patients after hip fracture surgery: a randomized controlled trial. J Pers Med.

[40] Kalron A, Tawil H, Peleg-Shani S, Vatine JJ (2018). Effect of telerehabilitation on mobility in people after hip surgery: a pilot feasibility study. Int J Rehabil Res.

[41] Latham NK, Harris BA, Bean JF, Heeren T, Goodyear C, Zawacki S, Heislein DM, Mustafa J, Pardasaney P, Giorgetti M, Holt N, Goehring L, Jette AM (2014). Effect of a home-based exercise program on functional recovery following rehabilitation after hip fracture: a randomized clinical trial. JAMA.

[42] Li CT, Hung GK, Fong KN, Gonzalez PC, Wah SH, Tsang HW (2022). Effects of home-based occupational therapy telerehabilitation via smartphone for outpatients after hip fracture surgery: a feasibility randomised controlled study. J Telemed Telecare.

[43] Pfeiffer K, Kampe K, Klenk J, Rapp K, Kohler M, Albrecht D, Büchele G, Hautzinger M, Taraldsen K, Becker C (2020). Effects of an intervention to reduce fear of falling and increase physical activity during hip and pelvic fracture rehabilitation. Age Ageing.

[44] Krichbaum K (2007). GAPN postacute care coordination improves hip fracture outcomes. West J Nurs Res.

[45] Langford DP, Fleig L, Brown KC, Cho NJ, Frost M, Ledoyen M, Lehn J, Panagiotopoulos K, Sharpe N, Ashe MC (2015). Back to the future—feasibility of recruitment and retention to patient education and telephone follow-up after hip fracture: a pilot randomized controlled trial. Patient Prefer Adherence.

[46] Breedveld-Peters JJ, Reijven PLM, Wyers CE, van Helden S, Arts JJC, Meesters B, Prins MH, van der Weijden T, Dagnelie PC (2012). Integrated nutritional intervention in the elderly after hip fracture. A process evaluation. Clin Nutr.

[47] Wyers CE, Reijven PLM, Breedveld-Peters JJL, Denissen KFM, Schotanus MGM, van Dongen MCJM, Eussen SJPM, Heyligers IC, van den Brandt PA, Willems PC, van Helden S, Dagnelie PC (2018). Efficacy of nutritional intervention in elderly after hip fracture: a multicenter randomized controlled trial. J Gerontol A Biol Sci Med Sci.

[48] Córcoles-Jiménez MP, Candel-Parra E, Del Egido-Fernández MÁ, Villada-Munera A, Moreno-Moreno M, Piña-Martínez AJ, Jiménez-Sánchez MD, Azor-García RJ (2021). Preventing functional urinary incontinence in hip-fractured older adults through patient education: a randomized controlled trial. J Appl Gerontol.

[49] Gardner MJ, Brophy RH, Demetrakopoulos D, Koob J, Hong R, Rana A, Lin JT, Lane JM (2005). Interventions to improve osteoporosis treatment following hip fracture. A prospective, randomized trial. J Bone Joint Surg Am.

[50] Di Monaco M, De Toma E, Gardin L, Giordano S, Castiglioni C, Vallero F (2015). A single postdischarge telephone call by an occupational therapist does not reduce the risk of falling in women after hip fracture: a randomized controlled trial. Eur J Phys Rehabil Med.

[51] Cheng KC, Lau KMK, Cheng ASK, Lau TSK, Lau FOT, Lau MCH, Law SW (2022). Use of mobile app to enhance functional outcomes and adherence of home-based rehabilitation program for elderly with hip fracture: a randomized controlled trial. Hong Kong Physiother J.

[52] Ortiz-Piña M, Molina-Garcia P, Femia P, Ashe MC, Martín-Martín L, Salazar-Graván S, Salas-Fariña Z, Prieto-Moreno R, Castellote-Caballero Y, Estevez-Lopez F, Ariza-Vega P (2021). Effects of tele-rehabilitation compared with home-based in-person rehabilitation for older adult's function after hip fracture. Int J Environ Res Public Health.

[53] Bedra M, Finkelstein J (2015). Feasibility of post-acute hip fracture telerehabilitation in older adults. Stud Health Technol Inform.

[54] Stevens S, Gallagher S, Andrews T, Ashall-Payne L, Humphreys L, Leigh S (2022). The effectiveness of digital health technologies for patients with diabetes mellitus: a systematic review. Front Clin Diabetes Healthc.

[55] von Huben A, Howell M, Howard K, Carrello J, Norris S (2021). Health technology assessment for digital technologies that manage chronic disease: a systematic review. Int J Technol Assess Health Care.

[56] Fahy N (2021). Use of digital health tools in Europe: before, during and after COVID-19. European Observatory on Health Systems and Policies.

[57] Dhingra D, Dabas A (2020). Global strategy on digital health. Indian Pediatr.

[58] Kim MY, Lee EJ (2019). Factors affecting self-care behavior levels among elderly patients with type 2 diabetes: a quantile regression approach. Medicina (Kaunas).

[59] Vincent C, Wegier P, Chien V, Kurahashi AM, Ginsburg S, Molla Ghanbari H, Wolfstadt JI, Cram P (2021). Qualitative evaluation of a novel educational tool to communicate individualized hip fracture prognostic information to patients and surrogates: my hip fracture (my-HF). Geriatr Orthop Surg Rehabil.

[60] Yadav L, Haldar A, Jasper U, Taylor A, Visvanathan R, Chehade M, Gill T (2019). Utilising digital health technology to support patient-healthcare provider communication in fragility fracture recovery: systematic review and meta-analysis. Int J Environ Res Public Health.

[61] Crotty M, Whitehead C, Miller M, Gray S (2003). Patient and caregiver outcomes 12 months after home-based therapy for hip fracture: a randomized controlled trial. Arch Phys Med Rehabil.

[62] Paul B, Saha I, Kumar S, Samim Ferdows SK, Ghose G (2015). Mobile phones: time to rethink and limit usage. Indian J Public Health.

[63] Hirsch KR, Wolfe RR, Ferrando AA (2021). Pre- and post-surgical nutrition for preservation of muscle mass, strength, and functionality following orthopedic surgery. Nutrients.

[64] Inoue T, Iida Y, Takahashi K, Shirado K, Nagano F, Miyazaki S, Takeuchi I, Yoshimura Y, Momosaki R, Maeda K, Wakabayashi H (2022). Nutrition and physical therapy: a position paper by the physical therapist section of the Japanese Association of Rehabilitation Nutrition (Secondary Publication). JMA J.

[65] Sanford JA, Griffiths PC, Richardson P, Hargraves K, Butterfield T, Hoenig H (2006). The effects of in-home rehabilitation on task self-efficacy in mobility-impaired adults: a randomized clinical trial. J Am Geriatr Soc.

