# Supplementary material for: Characteristics and Effects of Home-Based Digital Health Interventions on Functional Outcomes in Older Patients With Hip Fractures After Surgery: Systematic Review and Meta-Analysis
Source: J Med Internet Res. 2024 Jun 12;26:e49482. doi: 10.2196/49482 (PMC11208838; doi:10.2196/49482)
Supplement: Multimedia Appendix 2 [file jmir_v26i1e49482_app2.docx]

**Table S1.** PubMed search strategies assess to all databases.

| Final Query in PubMed (Last Accessed on January 3, 2023) | Item found |
| --- | --- |
| ("hip fractures"[MeSH Terms] OR ("hip"[All Fields] AND "fractures"[All Fields]) OR "hip fractures"[All Fields] OR ("hip"[All Fields] AND "fracture"[All Fields]) OR "hip fracture"[All Fields] OR ("femoral neck fractures"[MeSH Terms] OR ("femoral"[All Fields] AND "neck"[All Fields] AND "fractures"[All Fields]) OR "femoral neck fractures"[All Fields] OR ("femoral"[All Fields] AND "neck"[All Fields] AND "fracture"[All Fields]) OR "femoral neck fracture"[All Fields]) OR (("broken"[All Fields] AND "hip"[All Fields]) OR "broken hip"[All Fields]))  AND ("lancet digit health"[Journal] OR "eur heart j digit health"[Journal] OR "digit health"[Journal] OR ("digital"[All Fields] AND "health"[All Fields]) OR "digital health"[All Fields] OR ("telemedicine"[MeSH Terms] OR "telemedicine"[All Fields] OR "telemedicine s"[All Fields]) OR "telehealth"[All Fields] OR "ehealth"[All Fields] OR "e-Health"[All Fields] OR "mhealth"[All Fields] OR "m-health"[All Fields] OR (("mobile"[All Fields] AND "health"[All Fields]) OR "mobile health"[All Fields]) OR (("tele"[All Fields] AND "referral"[All Fields]) OR "tele referral"[All Fields]) OR (("virtual"[All Fields] AND "medicine"[All Fields]) OR "virtual medicine"[All Fields]) OR ("telemonitor"[All Fields] OR "telemonitored"[All Fields] OR "telemonitoring"[All Fields] OR "telemonitors"[All Fields]) OR ("telecommunications"[MeSH Terms] OR "telecommunications"[All Fields] OR "telecommunication"[All Fields]) OR ("telerehabilitation"[MeSH Terms] OR "telerehabilitation"[All Fields]) OR ("mobile applications"[MeSH Terms] OR ("mobile"[All Fields] AND "applications"[All Fields]) OR "mobile applications"[All Fields] OR ("mobile"[All Fields] AND "application"[All Fields]) OR "mobile application"[All Fields]) OR (("mobile"[All Fields] AND "app"[All Fields]) OR "mobile app"[All Fields]) OR (("smartphone"[All Fields] AND "apps"[All Fields]) OR "smartphone apps"[All Fields]) OR (("portable"[All Fields] AND "electronic"[All Fields] AND "apps"[All Fields]) OR "portable electronic apps"[All Fields]) OR ("smartphone"[MeSH Terms] OR "smartphone"[All Fields] OR "smartphones"[All Fields] OR "smartphone s"[All Fields]) OR ("telephone"[MeSH Terms] OR "telephone"[All Fields] OR "telephones"[All Fields] OR "telephoned"[All Fields] OR "telephonic"[All Fields] OR "telephonically"[All Fields] OR "telephoning"[All Fields]) OR ("phone s"[All Fields] OR "phoned"[All Fields] OR "phones"[All Fields] OR "phoning"[All Fields] OR "telephone"[MeSH Terms] OR "telephone"[All Fields] OR "phone"[All Fields]) OR (("lancet digit health"[Journal] OR "eur heart j digit health"[Journal] OR "digit health"[Journal] OR ("digital"[All Fields] AND "health"[All Fields]) OR "digital health"[All Fields]) AND ("technology"[MeSH Terms] OR "technology"[All Fields] OR "technologies"[All Fields] OR "technology s"[All Fields])) OR ("computability"[All Fields] OR "computable"[All Fields] OR "computating"[All Fields] OR "computation"[All Fields] OR "computational"[All Fields] OR "computations"[All Fields] OR "compute"[All Fields] OR "computed"[All Fields] OR "computer s"[All Fields] OR "computers"[MeSH Terms] OR "computers"[All Fields] OR "computer"[All Fields] OR "computes"[All Fields] OR "computing"[All Fields] OR "computional"[All Fields]) OR ("internet"[MeSH Terms] OR "internet"[All Fields] OR "internet s"[All Fields] OR "internets"[All Fields]) OR ("text messaging"[MeSH Terms] OR ("text"[All Fields] AND "messaging"[All Fields]) OR "text messaging"[All Fields]) OR ("multimedia"[MeSH Terms] OR "multimedia"[All Fields] OR "multimedium"[All Fields]) OR (("digital"[All Fields] OR "digitalisation"[All Fields] OR "digitalised"[All Fields] OR "digitalization"[All Fields] OR "digitalize"[All Fields] OR "digitalized"[All Fields] OR "digitalizer"[All Fields] OR "digitalizing"[All Fields] OR "digitally"[All Fields] OR "digitals"[All Fields] OR "digitization"[All Fields] OR "digitizations"[All Fields] OR "digitize"[All Fields] OR "digitized"[All Fields] OR "digitizer"[All Fields] OR "digitizers"[All Fields] OR "digitizes"[All Fields] OR "digitizing"[All Fields]) AND ("intervention s"[All Fields] OR "interventions"[All Fields] OR "interventive"[All Fields] OR "methods"[MeSH Terms] OR "methods"[All Fields] OR "intervention"[All Fields] OR "interventional"[All Fields])))  AND ("postoperative period"[MeSH Terms] OR ("postoperative"[All Fields] AND "period"[All Fields]) OR "postoperative period"[All Fields] OR ("post"[All Fields] AND "operative"[All Fields]) OR "post operative"[All Fields] OR ("post"[All Fields] AND ("surgery"[MeSH Subheading] OR "surgery"[All Fields] OR "surgical procedures, operative"[MeSH Terms] OR ("surgical"[All Fields] AND "procedures"[All Fields] AND "operative"[All Fields]) OR "operative surgical procedures"[All Fields] OR "general surgery"[MeSH Terms] OR ("general"[All Fields] AND "surgery"[All Fields]) OR "general surgery"[All Fields] OR "surgery s"[All Fields] OR "surgerys"[All Fields] OR "surgeries"[All Fields])) OR ("post"[All Fields] AND ("discharges"[All Fields] OR "discharging"[All Fields] OR "patient discharge"[MeSH Terms] OR ("patient"[All Fields] AND "discharge"[All Fields]) OR "patient discharge"[All Fields] OR "discharge"[All Fields] OR "discharged"[All Fields])) OR "post-discharge"[All Fields] OR ("postdischarge"[All Fields] OR "postdischarges"[All Fields]) OR ("subacute care"[MeSH Terms] OR ("subacute"[All Fields] AND "care"[All Fields]) OR "subacute care"[All Fields]) OR ("post"[All Fields] AND ("acute"[All Fields] OR "acutely"[All Fields] OR "acutes"[All Fields]) AND ("phase"[All Fields] OR "phase s"[All Fields] OR "phases"[All Fields])) OR (("post"[All Fields] AND "acute"[All Fields] AND "care"[All Fields]) OR "post acute care"[All Fields]) OR ("transitional care"[MeSH Terms] OR ("transitional"[All Fields] AND "care"[All Fields]) OR "transitional care"[All Fields]) OR ("transitional"[All Fields] AND "cares"[All Fields]) OR (("transition"[All Fields] AND "care"[All Fields]) OR "transition care"[All Fields]) OR ("transition"[All Fields] AND "cares"[All Fields]) OR (("home"[All Fields] AND "transition"[All Fields]) OR "home transition"[All Fields]) OR (("home"[All Fields] AND "transitions"[All Fields]) OR "home transitions"[All Fields]) OR (("intermediate"[All Fields] OR "intermediated"[All Fields] OR "intermediately"[All Fields] OR "intermediates"[All Fields]) AND "care"[All Fields]) OR (("intermediate"[All Fields] OR "intermediated"[All Fields] OR "intermediately"[All Fields] OR "intermediates"[All Fields]) AND ("care s"[All Fields] OR "cared"[All Fields] OR "carefulness"[All Fields] OR "cares"[All Fields] OR "empathy"[MeSH Terms] OR "empathy"[All Fields] OR "caring"[All Fields])) OR ("home-based"[All Fields] AND "care"[All Fields]) OR ("rehabilitant"[All Fields] OR "rehabilitants"[All Fields] OR "rehabilitate"[All Fields] OR "rehabilitated"[All Fields] OR "rehabilitates"[All Fields] OR "rehabilitating"[All Fields] OR "rehabilitation"[MeSH Terms] OR "rehabilitation"[All Fields] OR "rehabilitations"[All Fields] OR "rehabilitative"[All Fields] OR "rehabilitation"[MeSH Subheading] OR "rehabilitation s"[All Fields] OR "rehabilitational"[All Fields] OR "rehabilitator"[All Fields] OR "rehabilitators"[All Fields]) OR ("fall"[All Fields] AND ("assess"[All Fields] OR "assessed"[All Fields] OR "assessement"[All Fields] OR "assesses"[All Fields] OR "assessing"[All Fields] OR "assessment"[All Fields] OR "assessment s"[All Fields] OR "assessments"[All Fields])) OR ("nutritional support"[MeSH Terms] OR ("nutritional"[All Fields] AND "support"[All Fields]) OR "nutritional support"[All Fields] OR ("nutrition"[All Fields] AND "support"[All Fields]) OR "nutrition support"[All Fields]) OR (("injuries"[MeSH Subheading] OR "injuries"[All Fields] OR "wounds"[All Fields] OR "wounds and injuries"[MeSH Terms] OR ("wounds"[All Fields] AND "injuries"[All Fields]) OR "wounds and injuries"[All Fields] OR "wound s"[All Fields] OR "wounded"[All Fields] OR "wounding"[All Fields] OR "woundings"[All Fields] OR "wound"[All Fields]) AND "care"[All Fields])) | 1127 |

**Table S2.** The definition of each category in 3 dimensions.

| **Category** | **Definition** |
| --- | --- |
| Purpose and Content-based classification | |
| 1.Education | DHIs that provide health-related education or information, such as online courses, webinars, or informational videos. |
| 2.Tele-Rehabilitation | DHIs that provide remote rehabilitation services by healthcare providers |
| 3.Communication and feedback | DHIs that support communication between patients and healthcare providers and provide some feedback to user, such as secure messaging, and video consultations. |
| 4.Behavioral and lifestyle interventions/Health coaching | DHIs that target behavior change related to a health condition or disease. Examples include goal setting, encourage healthy behaviors, or provide personalized health coaching. |
| 5.Remote monitoring | DHIs that monitor vital signs or physical activity of participants |
| Mode of delivery | |
| 1.Web-based and software | online website or software that allow patients to access their personal health information conveniently from anywhere with an internet connection |
| 2.Telephone call | the use of phone calls by healthcare providers to deliver self-care support and/or management |
| 3.Mobile applications | technology-based solutions that provide support, delivery, and promotion of care through mobile devices such as smartphones, wearables, and health tracking apps. |
| 4.Sensor-based technology | devices or systems that use sensors to collect and transmit data about a patient's health or environment to healthcare providers for analysis and intervention |
| Healthcare providers (HCPs) | |
| 1.Occupational therapists (OT) | HCPs who focus on helping patients perform daily tasks more easily, improving their fine and gross motor skills, and making their home environment more optimal for their everyday life. |
| 2.Physical therapists or Physiotherapists (PT) | HCPs who focus on helping improve patients’ movement, mobility, and function by using a variety of exercises, stretches, or other physical activities. |
| 3.Physicians | HCPs who have role of diagnose, treat and prevent illness, disease, injury, and other physical and mental impairments and maintain general health including general practitioner, specialist doctor |
| 4.Nurses | HCPs who provide treatment, support and care services for patients who need nursing care |
| 5.Dieticians | HCPs or nutritionists who assess, plan and implement programs to enhance the impact of food and nutrition on human health |
| 6.Multidisciplinary teams | a diverse group of HCPs working together |


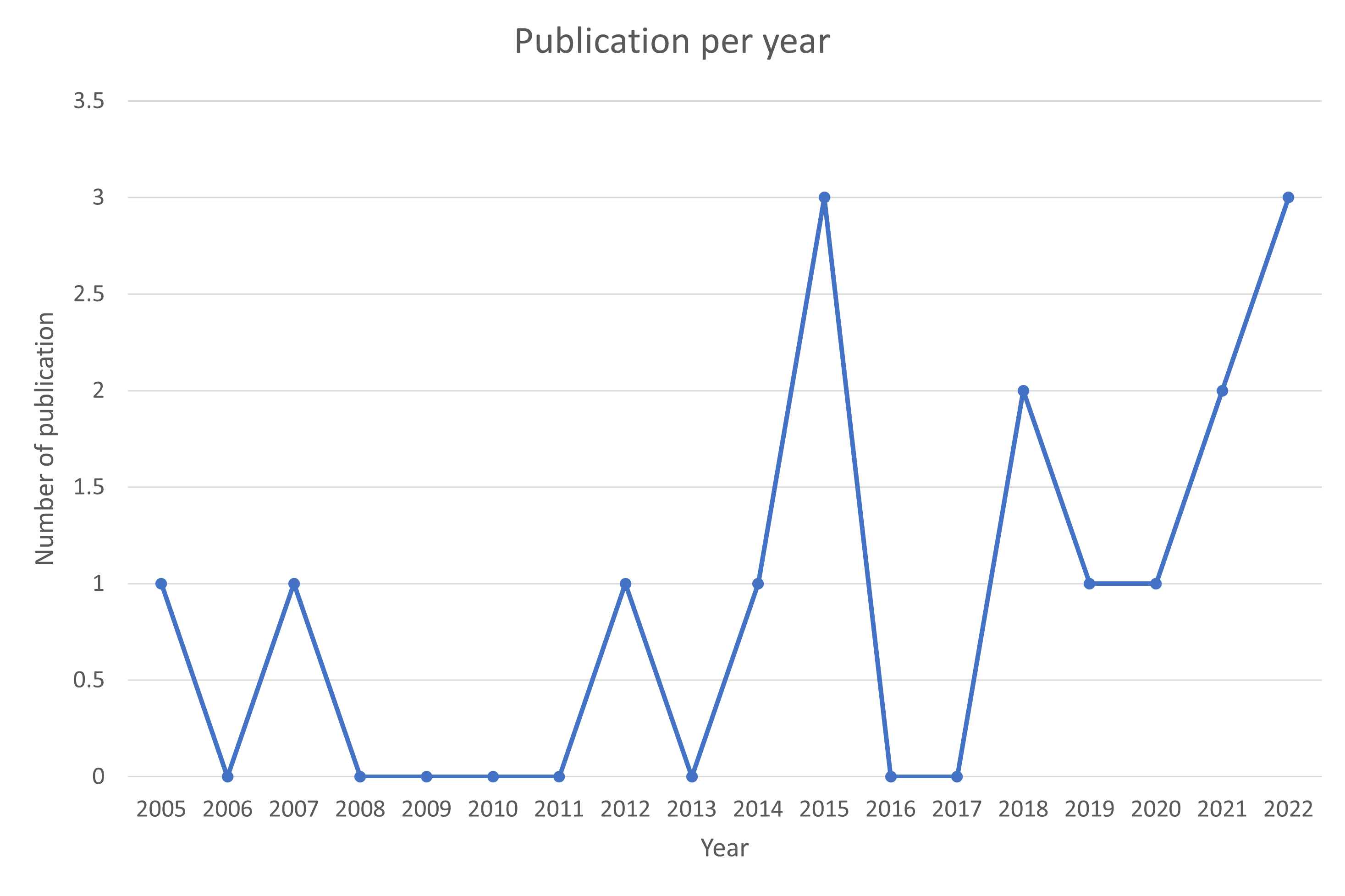


**Figure S1.** The total number of publications for each year.

**Table S3.** Other information of included studies.

| **First Author, Year of publication** | **Country** | **Intervention** | **Date of DHIs** | **Time to measure outcome** | **Other outcomes** |
| --- | --- | --- | --- | --- | --- |
| Gardner, 2005 | USA | - Educational intervention with discussion 5 questions regarding osteoporosis treatment  - single telephone call to remind questions during a follow-up | Once at 6 weeks postoperatively | 6 months | osteoporosis addressed by their primary physician.  I-group 42%, C-group 19%  (*P*= .036) |
| Krichbaum, 2007 | USA | Nursing post-acute care coordination intervention  - first month: face to face then face to face or telephone until 6 months  - health assessment | 1^st^ month once a week, then twice per week until 6 months | -baseline  -1 month  -3 months  -6 months  -12 months | no significant differences in mood (depression), health, or living situation between groups |
| Breedveld-Peters, 2012 | Netherland | Dietetic counseling (telephone call 5 visits and face-face 5 visits) and ONS for 3 months | 3,4,5,8,10 weeks after discharge | 3 months | - Adherence of staff to protocol: 83% all 10 visits  - nutrition counseling was complete in 91% |
| Latham, 2014 | USA | functionally oriented exercises, home exercise program 3 times/week for 6 months  - monthly telephone call  - DVD version of the program | for 6 months | -baseline  -End of 6 months intervention  -9 months | -mean AM-PAC daily activity between group difference 3.5 [95% CI (0.9-6.0), *P*=.03]  -in multiple imputation mean AM-PAC mobility between group difference not significant |
| Di Monaco, 2015 | Italy | - multidisciplinary rehabilitation program during hospitalization  - single telephone call to reinforce the targeted recommendations for fall prevention | once at a median of 18 days after discharge | 6 months | - adherence to fall prevention recommendations (mean difference 3.9, [95% CI  (-3.4 to 11.3), *P*=.29] |
| Bedra, 2015 | USA | Post comprehensive telerehabilitation system to support individualized exercise program using home Automated Telemanagement. (HAT)  - tailored feedback  - multimedia education individualized to patient’s need | for 30 days | End of 30 days intervention | Significantly improve quality of life (SF-36) in physical functioning, role limitations due to physical health problems, social Functioning, health Transition |
| Langford, 2015 | Canada | Educational (1-hour in-hospital session) plus 5 post discharge telephone call coaching | for 4 months | -baseline  -End of 4 months intervention | - recruitment 42%, (success 30%), retention rate at 4 months 90%  - no differences between groups for health measures (quality of life) |
| Kalron, 2018 | Israel | home based telerehabilitation: video clips of common rehabilitation exercises focusing on the lower limbs, 40-50 min/session | 3sessions/week for 6 weeks | -baseline  -End of 6-weeks intervention  -4 weeks post intervention | During follow-up, I-group continued to improve in all outcome measures |
| Wyers, 2018 | Netherland | Intensive nutritional intervention  - 10 counselling: 2 sessions in hospitalization and 8 sessions of weekly dietetic consultation (3 face to face, 5 telephone calls)  - energy-protein-enriched diet and ONS for 3 months | 8 sessions for 3 months | -End of 3 months intervention  -6 months  -1 year  -5 years | - median total LOS 34 d (range 4-185) in I-group, 35.5 d (range 3-183) in C-group (*P*=.80, adjHR 0.98 (95%CI 0.68-1.41)  - intervention improved nutritional intake/status at 3 months, but not at 6 months |
| Pol, 2019  (3-arm RCT) | Netherland | - CBT based occupational therapy (weekly session coaching of skilled nursing facility, 4 home visits,  4 telephone consultation)  - sensor monitoring (physical activity monitor, motion sensors) | for 3 months | -baseline  -1 month  -4 months  -6 months | no significant differences in daily functioning between CBT-based OT and care as usual. |
| Pfeiffer, 2020 | Germany | - 8 individual sessions during inpatient rehabilitation  - additional support via 1 home visit, 4 telephone calls | 4 times in 2 months | -admissionT0  -before discharge: T1  -3 months after discharge: T2 | - I-group significant improvement in short FES-I, but no difference in total daily walking duration (*P* = .688, d = 0.07) at T2 compared with C-group |
| Ortiz-Piña, 2021 | Spain | @ctivehip: multidisciplinary  tele-rehabilitation program supervised by family caregivers, 50-60 minute/ session, 5 session/week in 2 online components:  3 exercise sessions + 2 OT sessions  - videoconference (if need) | 5 session/week for 12 weeks | -baseline  -1 month  -End of 3 months intervention | - |
| Córcoles-Jiménez, 2021 | Spain | -Urinary habit training (education): in hospital stay  -Telephonic reinforcement to remind the recommended activities, repeating list of activities. | between the 7th and 10th day after discharge. | 3 months  6 months | - relative risk 0.52 (0.3-0.9), NNT 4  - mean of urinary incontinent (UI) episode 1.8 in I-group, 0.54 in C-group (*P* =.007) |
| Zhang, 2022 | China | Home based telerehabilitation.  -personalized rehabilitation programs, video, health knowledge  - remote monitoring vital sign, assessment &guidance  - appointment for consultation | for 3 months | -1month post-intervention  -3-month post-intervention | - |
| Cheng, 2022 | Hong Kong | Mobile app in delivering home-based rehabilitation program.  - briefing session before discharge  - perform exercise along exercise video in app | for 2 months | -baseline  -1month during home visit  -End of 2 months intervention | - I-group showed higher exercise adherence than the control group in the first month (*P* = .03)  - no difference in modified caregiver strain index (M-CSI) at 1 and 2 months |
| Li, 2022 | Hong Kong | home program using the Caspar Health e-system.  - tailor-made Tele-rehabilitation program through e-system calendar  - video, pictures, written and verbal instructions show on app  - patient feedback and OT update program | for 3 weeks | -baseline  -End of 3-week intervention  -3 weeks post intervention | - I-group significant improvement in fall efficacy (MFS, *P*=.002) at post-intervention and follow up  - no significant differences in pain visual analogue scale (pain VAS), FES |

^a^ Non-RCT study, ^b^ Pol, 2019 was only one studies in 3-arm RCT, others were studies in 2-arm RCT

**Table S4.** GRADE for quality of evidence profile.

Patients or population: older adults with hip fractures

Settings: home

Intervention: home-based DHIs

Comparison: usual care

| Outcome | Number of participants (studies) | Risk of bias | Inconsistency | Indirectness | Imprecision | Publication Bias | Certainty of the evidence (GRADE) |
| --- | --- | --- | --- | --- | --- | --- | --- |
| TUG | 176  (3 RCTs, 1Non-RCT) | Not serious | Not serious | Not serious | Serious | Not applicable | ⊕⊕⊕, moderate |
| SPPB | 404  (3 RCTs, 1Non-RCT) | Not serious | Not serious | Serious | Not  serious | Not applicable | ⊕⊕⊕, moderate |
| FIM | 113  (1 RCTs, 1Non-RCT) | Not serious | Not serious | Not serious | Serious | Not applicable | ⊕⊕⊕, moderate |
